# Research Progress in the Modification of Quercetin Leading to Anticancer Agents

**DOI:** 10.3390/molecules22081270

**Published:** 2017-07-29

**Authors:** Alessandro Massi, Olga Bortolini, Daniele Ragno, Tatiana Bernardi, Gianni Sacchetti, Massimo Tacchini, Carmela De Risi

**Affiliations:** 1 Dipartimento di Scienze Chimiche e Farmaceutiche, Università di Ferrara, Via Luigi Borsari 46, I-44121 Ferrara, Italy; alessandro.massi@unife.it (A.M.); olga.bortolini@unife.it (O.B.); daniele.ragno@unife.it (D.R.); tatiana.bernardi@unife.it (T.B.); 2 Dipartimento di Scienze della Vita e Biotecnologie, Sezione di Botanica Applicata, Piazzale Luciano Chiappini 3, I-44123 Ferrara, Italy; gianni.sacchetti@unife.it (G.S.); massimo.tacchini@unife.it (M.T.)

**Keywords:** quercetin, quercetin derivatives, methoxyflavones, anticancer, cell proliferation, cytotoxicity, multi-drug resistance (MDR)

## Abstract

The flavonoid quercetin (3,3′,4′,5,7-pentahydroxyflavone) is widely distributed in plants, foods, and beverages. This polyphenol compound exhibits varied biological actions such as antioxidant, radical-scavenging, anti-inflammatory, antibacterial, antiviral, gastroprotective, immune-modulator, and finds also application in the treatment of obesity, cardiovascular diseases and diabetes. Besides, quercetin can prevent neurological disorders and exerts protection against mitochondrial damages. Various in vitro studies have assessed the anticancer effects of quercetin, although there are no conclusive data regarding its mode of action. However, low bioavailability, poor aqueous solubility as well as rapid body clearance, fast metabolism and enzymatic degradation hamper the use of quercetin as therapeutic agent, so intense research efforts have been focused on the modification of the quercetin scaffold to obtain analogs with potentially improved properties for clinical applications. This review gives an overview of the developments in the synthesis and anticancer-related activities of quercetin derivatives reported from 2012 to 2016.

## 1. Introduction

**Figure 1:**
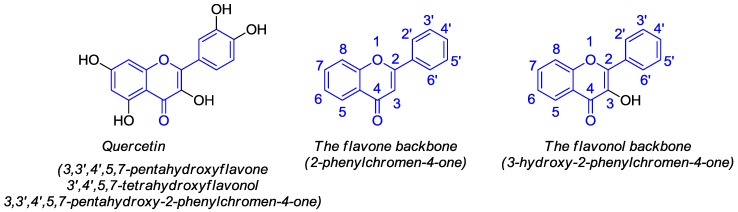
Structure of quercetin and representation of flavone and flavonol backbones.

Quercetin, namely 3,3′,4′,5,7-pentahydroxyflavone (3′,4′,5,7-tetrahydroxyflavonol or 3,3′,4′,5,7-pentahydroxy-2-phenylchromen-4-one) (Figure 1), belongs to the flavonol (3-hydroxyflavone) group of polyphenolic compounds known as flavonoids.

**Figure 2:**
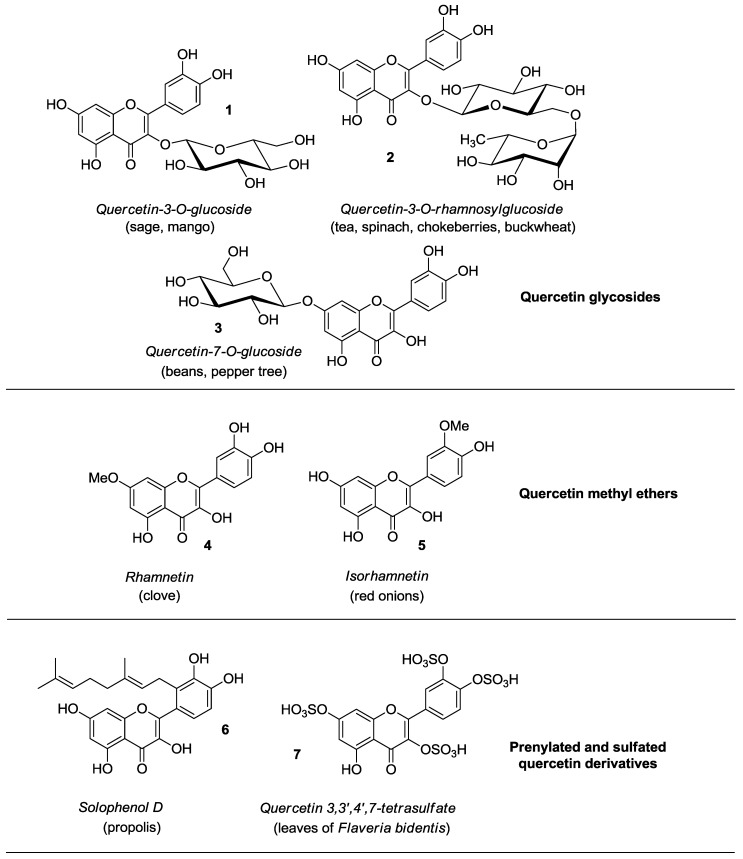
Structures of quercetin derivatives **1**–**7** along with their occurrence in food and plants.

Quercetin is abundantly present in diverse plant materials (leaves, grains, fruits, and vegetables) as well as in common foods and drinks [1,2,3], with onions, apples, berries, broccoli, tea, and red wine serving as typical examples. In plants, quercetin can exist in either free (aglycone) or bounded form, mainly with carbohydrates (quercetin glycosides) and alcohols, mostly methanol (quercetin methyl ethers), while less frequently occurring are quercetin derivatives featuring prenyl and sulfate substituents [4]. Some representative quercetin conjugated compounds (**1**–**7**) are depicted in Figure 2.

With particular regard to quercetin glycosides, either monosaccharides or disaccharides are generally attached at the C-3 position of quercetin, however glycosylation of other hydroxyl groups may occur. For example, quercetin 3-*O*-glucoside **1** was found in sage and mango [5,6], with the latter containing quercetin 3-*O*-galactoside, rhamnoside, and xyloside too. In addition, quercetin 3-*O*-rhamnoside has been detected in spinach [7], hot pepper [8], and olives [9]. Quercetin 3-*O*-rhamnosylglucoside (rutin, **2**) is present in tea [10], spinach [7], chokeberries [11], and buckwheat [12]. Instead, quercetin 7-*O*-glucoside **3** occurs in beans and aerial parts of pepper tree [13,14], whereas quercetin 3-*O*-rhamnoside-7-*O*-glucoside is a typical component of pepper [8].

Once ingested, quercetin glycosides are hydrolyzed, and the released aglycone is adsorbed and metabolized giving rise to glucuronidated, methylated, and sulfated derivatives, i.e., quercetin-3-*O*-glucuronide, 3′-*O*-methyl-quercetin (isorhamnetin, **5**), isorhamnetin 3-*O*-glucuronide, and quercetin-3′-*O*-sulfate, which enter the bloodstream [15]. Generally, neither free quercetin or its parent glycosides are detected in the plasma, wherein quercetin exists just in conjugate form.

Several decades ago quercetin attracted considerable attention as it was revealed to produce DNA mutations in bacteria. This result anticipated it as a cancer-causing agent, however inconclusive animal research as well as little evidence in humans did not seem to support this idea. On the contrary, recent years have evidenced several possible beneficial effects of quercetin, included its role in prevention and therapy of cancer [16].

In fact, quercetin functions as antioxidant, radical-scavenging, anti-inflammatory, antibacterial, antiviral, gastroprotective, immune-modulator, and is used in the treatment of obesity and cardiovascular diseases [15,17,18,19,20,21,22]. Moreover, quercetin may find application in anti-diabetic research [23], and is involved in the prevention of neurological disorders due to its neuroprotective effects [24]. Recently [25], it has been postulated that quercetin exerts protection against mitochondrial damages as a result of its ability to interact with several mitochondrial processes that are supposed to affect cells and tissues.

Due to its lipophilic nature, quercetin passes with ease through cell membranes and plays pleiotropic roles in triggering diverse intracellular routes implicated in chemoprevention (e.g., apoptosis, cell cycle, detoxification, antioxidant replication, cell invasion, angiogenesis) [26,27,28]. Nonetheless, there is no final proof regarding the anticancer mode of action of quercetin, with in vitro experiments showing that it could suppress multiple oncogenic signaling pathways [29,30]. On the contrary, it has been clearly demonstrated that anticancer effects of quercetin are site-specific [31].

Yet still, low bioavailability and poor solubility in water [32], together with rapid body clearance, fast metabolism, and enzymatic degradation, hamper to a great extent the clinical application of quercetin as an anticancer drug. Furthermore, quercetin has been shown to have in vitro toxic effects on normal human cell lines thereby setting limits to its possible in vivo use [33]. Speaking of which, intensive studies have been carried out on the pro-oxidant properties of quercetin and its metabolic conversion into potentially toxic quinones due to the presence of the catechol moiety [34]. In order to obtain quercetin analogs with improved properties for potential employment in cancer management, many synthetic efforts have been invested over the past ten years, and a water-soluble glycine carbamate ester quercetin prodrug (QC12) entered pre-clinical and clinical studies [35]. Regrettably, so far, no additional information about clinical development of this compound could be found in the literature.

The progresses in the synthesis and biological evaluation of quercetin derivatives as possible anticancer agents have been reviewed in 2009 by Hirpara et al. [36]. Since then, profuse research studies have been conducted on this topic, however no other literature survey has been hitherto reported, to the best of our knowledge. That being so, an overview of recent developments underlying the anticancer potential of synthetic quercetin derivatives is likely to be needed to provide an up-to-date picture of this research area.

In this review article, we gathered the diverse data published between 2012 and 2016, with attention being exclusively focused on compounds obtained using quercetin as the starting material. Among various anticancer-related effects of quercetin analogs, we selected activities against cancer cell lines as the exclusive focus of our manuscript. SciFinder database (Chemical Abstracts Service, Columbus, OH, USA) has been used as the literature source and papers in languages other than English have been excluded.

## 2. Synthesis and Anticancer-Related Activities of Quercetin Derivatives

**Figure 3:**
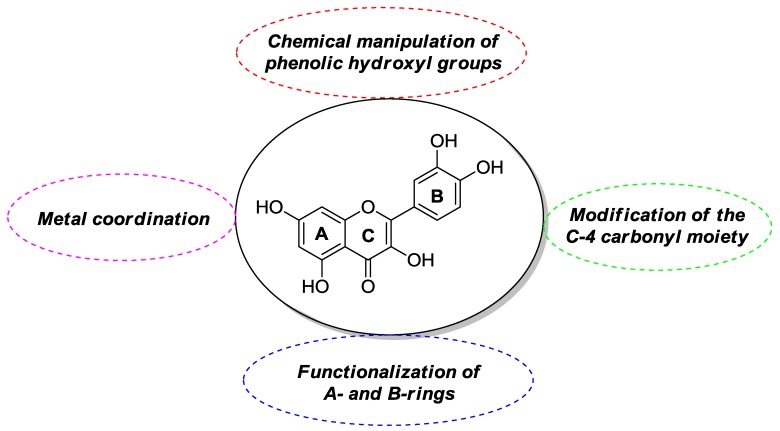
General methodologies towards modified forms of quercetin.

Quercetin derivatives with desirable properties for possible anticancer applications have been obtained through different synthetic routes. These include chemical manipulation of phenolic hydroxyl groups, possibly in combination with modifications at the C-4 carbonyl residue, functionalization of A- and B-rings, and metal coordination (Figure 3).

**Figure 4:**
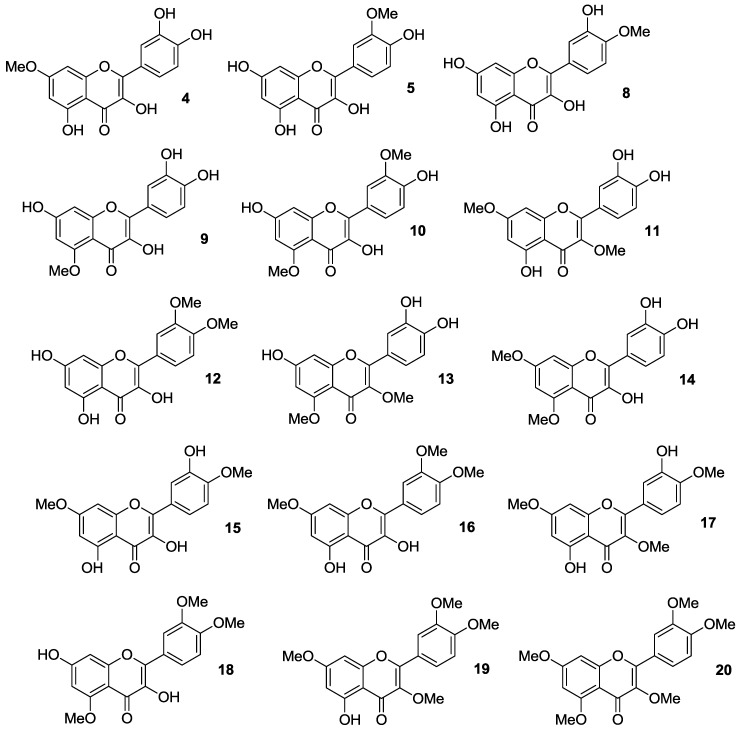
Structures of *O*-methylated quercetin derivatives (**4**), (**5**), and (**8**–**20**).

**Figure 5:**
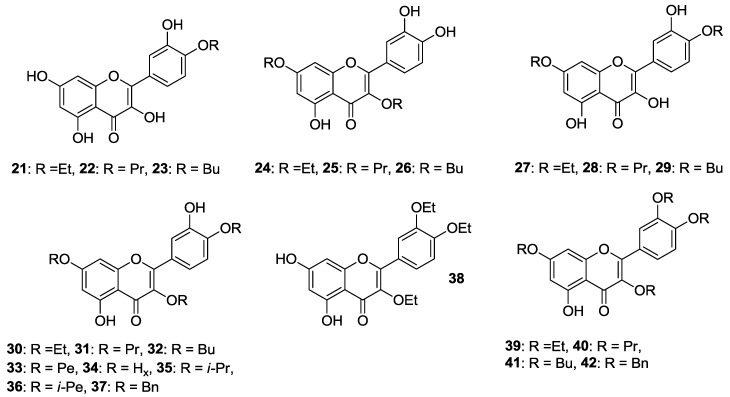

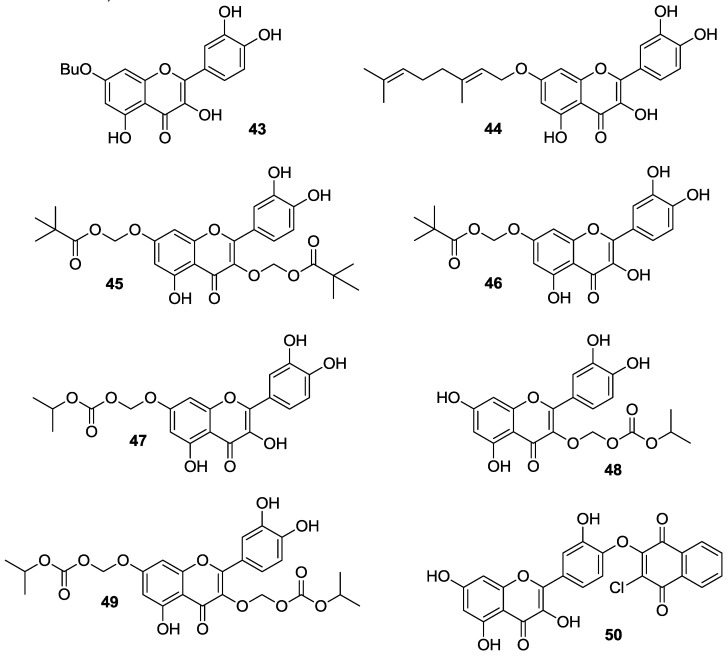
Structures of *O*-alkylated quercetin derivatives (**21**–**50**). Abbreviations: Et: ethyl, Pr: propyl, Bu: butyl, Pe: pentyl, H_x_: hexyl, *i*-Pr: isopropyl, *i*-Pe: isopentyl, Bn: benzyl.

**Figure 6:**
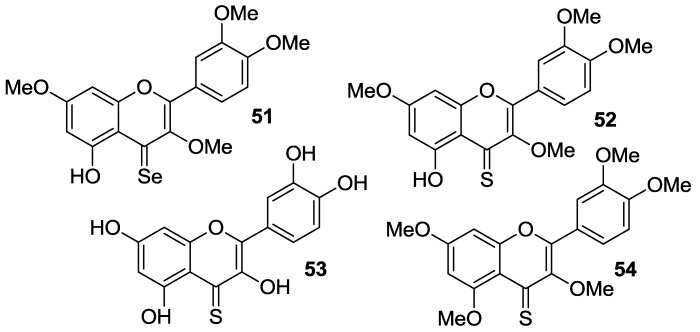
Structures of quercetin derivatives **51**–**54** obtained by manipulation of C-4 carbonyl moiety.

**Figure 7:**
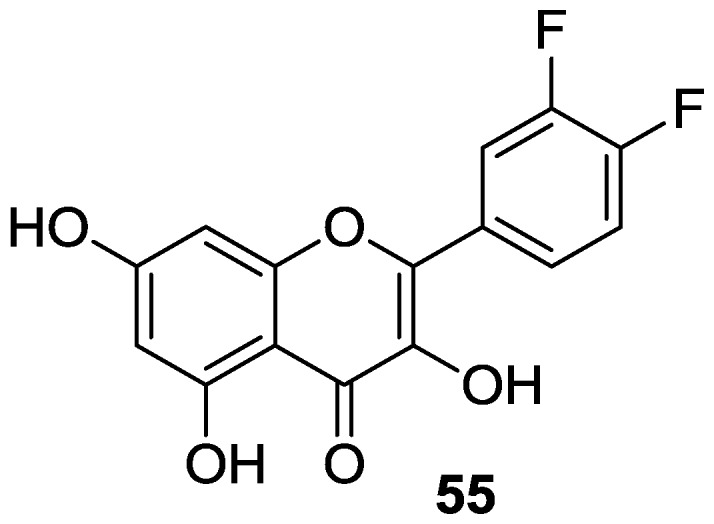
Structure of quercetin derivative **55** obtained via replacement of catecholic hydroxyl groups.

**Figure 8:**
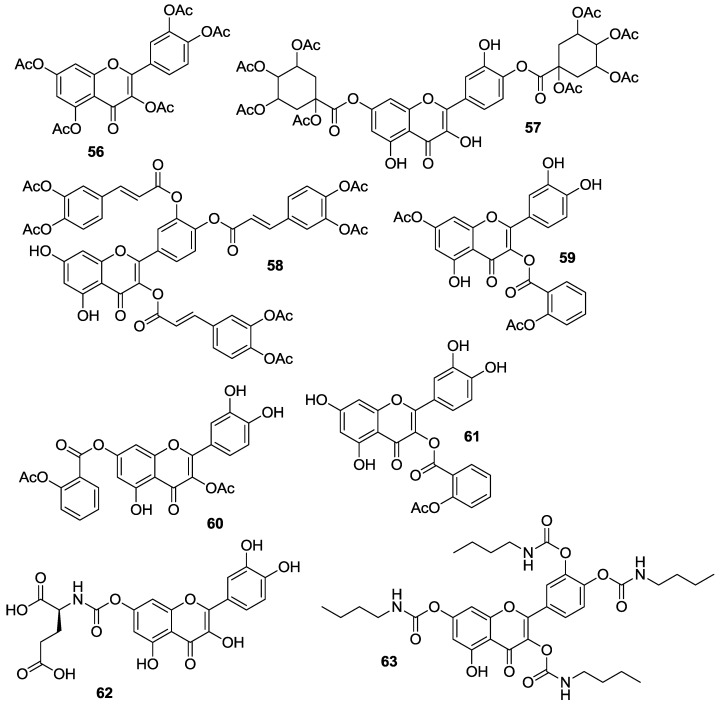
Structures of *O*-acylated quercetin derivatives **56**–**63**.

**Figure 9:**
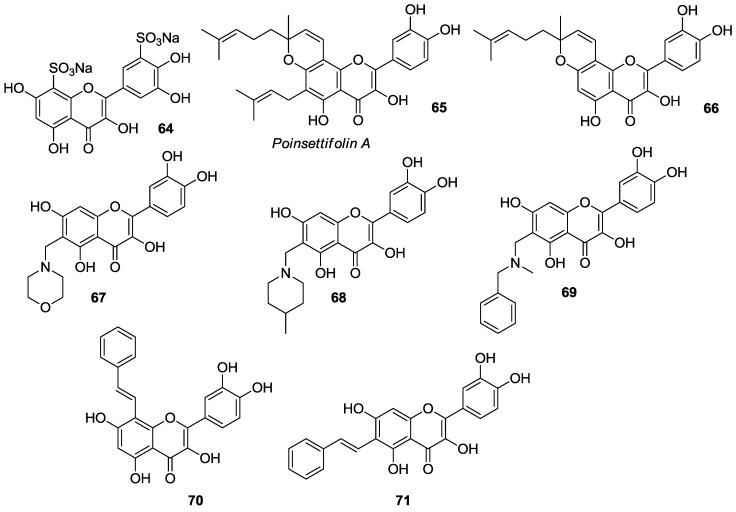
A- and B-ring functionalized quercetin derivatives **64**–**71**.

The collected literature material has been organized in subsections according to the way the quercetin analogs have been synthesized. Thus, compounds obtained by elaboration of either the phenolic hydroxyl groups or the C-4 carbonyl moiety are discussed in Section 2.1, while Section 2.2 focuses on species arising from functionalization of A- and B-rings. Finally, Section 2.3 describes quercetin-based metal complexes. For the sake of clarity, in all cases we chose to provide just a brief description of the synthetic details to give as much information as possible about biological activities. Accordingly, Figure 4, Figure 5, Figure 6, Figure 7, Figure 8, Figure 9 and Figure 11 exclusively show the structures of the discussed quercetin derivatives, with the synthetic schemes depicting their preparation being omitted.

### 2.1. Chemical Modification of Phenolic Hydroxyl Groups and/or C-4 Carbonyl Moiety

Phenolic hydroxyl groups of quercetin have been mostly manipulated by etherification (*O*-alkylation) and esterification (*O*-acylation), with the *O*-alkylation strategy being possibly accompanied by conversion of the C-4 carbonyl group into the corresponding thiocarbonyl or selenocarbonyl functions. Also, interchange of catecholic hydroxyl groups with bioisosteric moieties has been developed.

#### 2.1.1. *O*-Alkylation

It has been reported that insertion of methoxy groups into a flavone molecule results in metabolically more stable derivatives with increased solubility, bioavailability and cancer cell antiproliferative activity, as well as reduced toxic side-effects [37]. This information inspired several studies on the etherification of quercetin with either methyl or other alkyl groups in order to investigate their effect on either physico-chemical or anticancer-related properties.

Thus, quercetin was converted into a series of monomethylated (**4**,**5**,**8**,**9**), dimethylated (**10**–**15**), trimethylated (**16**–**18**), tetramethylated (**19**), and pentamethylated (**20**) derivatives [38,39,40,41,42], which are shown in Figure 4.

Synthetically, the mono-protected compounds were prepared by suitable protection/deprotection steps of the phenolic hydroxyl groups in quercetin, with methyl iodide/K_2_CO_3_ system in *N*,*N*-dimethylformamide (DMF) being conveniently used at the time of installing the methyl ether moiety. Conversely, direct treatment of the flavonol starting material with methyl iodide and potassium carbonate in either DMF or acetone was carried out to yield the di-, tri- tetra- and penta-functionalized analogs. 

In early studies [38,39], it was found that 3,3′,4′,7-tetra-*O*-methylated quercetin **19** and 3,3′,4′,5,7-penta-*O*-methylated quercetin **20** could represent potential anti-multidrug resistance (MDR) agents due to their ability to influence the effects of breast cancer resistance protein (BCRP), which is known to determine resistance in cancer cells. Importantly, both 3′,4′-OMe substitution and the presence of 5-OH group were essential for optimum BCRP inhibition, whereas this activity decreased upon methylation of C-5 phenolic hydroxyl group.

In particular, investigations in Madin-Darby canine kidney (MDCK) BCRP cells evidenced that **19** and **20** were able to inhibit BCRP as a result of Hoechst 33342 and pheophorbide A accumulation, contrary to quercetin, which gave no inhibitory effect (Table 1) [39].

Later, Shi et al. prepared the *O*-methylated compounds **4**, **5** and **8**–**20**, and evaluated their ability to inhibit cancer cell growth using a high-throughput screening (HTS) approach in an in vitro human disease-oriented cancer cell line, including melanoma (LOX-IMVI and M14), neck and head (M4E), cervical (HeLa), human breast cancer (SKBR) as well as human lung cancers (A549, H157, H460, 1792, 1944, H266, H522, Hop62, 1299, 292G, and Calu1) [40,41]. These investigations demonstrated that selective masking of the phenolic hydroxyl groups in quercetin is pivotal in determining antiproliferative activity. As a rule of thumb, it was possible to maintain inhibitory effects against all the cancer cell lines by methylation at the 4′-OH and/or 7-OH positions, while the coexistence of 3′- and 4′-OMe groups improved activity. Also, additional introduction of a methoxy moiety may enhance the inhibition of cancer cell growth, with 3′,4′,7-trimethoxyquercetin (**16**) being more potent than 3′,4′-dimethoxyquercetin (**12**).

The antiproliferative action of 3,7-*O*-dimethylquercetin (**11**), 3,4′,7-*O*-trimethylquercetin (**17**), and 3,3′,4′,7-*O*-tetramethylquercetin (**19**) against human androgen-refractory (DU-145 and PC-3) and androgen-sensitive (LNCaP) prostate cancer cell lines were examined as well [42], showing that methylation barely determined a weak enhancement of activity compared to parent quercetin.

Besides, the preparation of 4′-*O*-monoalkylated (**21**–**23**), 3,7-*O*-dialkylated (**24**–**26**), 4′,7-*O*-dialkylated (**27**–**29**), 3,4′,7-*O*-trialkylated (**30**–**37**), 3,3′,4′-*O*-trialkylated (**38**), and 3,3′,4′,7-*O*-tetraalkylated (**39**–**42**) derivatives of quercetin (Figure 5) was achieved in the same way as the quercetin methyl ether compounds [41,42]. Ensuing in vitro biological evaluation by the abovementioned HTS method lead Shi et al. to demonstrate that cancer cell growth inhibitory activities were retained when etherification of 3-OH and 4′-OH was carried out using the long propyl chain or the short ethyl one, respectively [41]. On the contrary, introduction of two *n*-butyloxy moieties into the 3,7 or 4′,7 sites enhanced the antiproliferative action.

Speaking of these studies, cytotoxicity data of the most representative *O*-alkylated quercetin derivatives are listed in Table 2.

With particular regard to human prostate cancer cells, Al-Jabban et al. concluded that antiproliferative activity strongly depended on either length or steric hindrance of the introduced alkyl chain [42]. Indeed, cancer cell growth greatly dropped when linear long or bulky alkyl groups were simultaneously introduced into C-3, C-4′ and C-7 hydroxyl groups, as observed for compounds (**31**–**34**) and (**35**,**36**), respectively. On the other hand, the derivative (**30**) appended with the short, linear ethyl group showed a slightly increased activity, similarly to the corresponding methyl analog (**17**, Figure 4). However, no significant change in activity was detected for 3,3′,4′-*O*-triethylquercetin (**38**). Importantly, the potency of 3,7-*O*-dialkylated derivatives (**24**–**26**) was 2–11 times higher than quercetin (Table 3), with this behaviour being also observed for the corresponding dimethylated compound (**11**, Figure 4).

It should be mentioned that the structures of the 4′,7-*O*-dialkylquercetins reported by Shi et al. [41] have been found to be wrong by heteronuclear multiple bond correlation (HMBC) nuclear magnetic resonance (NMR) experiments, and were corrected as the corresponding 3,7-*O*-dialkylated isomers [42].

A recent work by Khan and coworkers evidenced that 3,4′,7-*O*-triethylquercetin (**30**, Figure 5) was able to inhibit cell proliferation in colon (HCT-116) cancer cells (IC_50_ = 50 μM, 24 h incubation). Moreover, it behaved as apoptosis-inducer in the same cancer cell line without affecting normal cells growth [43]. It is worthwhile pointing out that **30** is supposed to take action through endoplasmic reticulum (ER) stress and a mitochondria-mediated pathway.

A three-step procedure involving peracetylation of quercetin followed by alkylation with a suitable alkyl chloride and base-mediated deacetylation gave access to 7-*O*-butylquercetin **43** and 7-*O*-geranylquercetin **44**, which are shown in Figure 5 [44,45]. These compounds showed a moderate solubility (180 μM) in Dulbecco’s modified eagle medium (DMEM) [45], and exhibited much stronger antiproliferative effects than quercetin in estrogen receptor-positive human breast cancer cell line (MCF-7), likely due to their better accumulation capability [44]. More precisely, the proliferation inhibitory activity of **43** and **44** depended on their apoptosis-inducing effects which were anyhow higher than those of quercetin. In this regard, it was demonstrated that the apoptotic process of MCF-7 cells occurred through a caspase-independent Endonuclease G (Endo G)-induced mitochondrial route, unlike quercetin.

It is worthy of note that compounds **43** and **44** did not affect normal breast epithelial (MCF-10A) cells and were also effective in estrogen receptor-negative MDA-MB-231 breast cancer cells. Furthermore, **43** and **44** were proposed to possess reversal activities on MDR cancer cells, but no evidence in support of this hypothesis was furnished. 

Further studies revealed that **44** had strong cytotoxicity on human colon (CaCo-2), human lung (NCI-H446 and A549) as well as human gastric (MGC-803 and SGC-7901) cancer cells thereby revealing potential antiproliferative properties [45]. In all cases, the observed activity proved to be higher as compared to quercetin. For the sake of clarity, the most relevant biological data regarding compounds **43** and **44** are detailed in Table 4.

In order to extend previous results on quercetin conjugates bearing a pivaloxymethyl (POM) promoiety at the 3 or the 7 position [46], the 3,7-bis-*O*-pivaloxymethyl (POM) quercetin (**45**, Figure 5) was prepared by sequential K_2_CO_3_-promoted alkylation of quercetin diphenylmethylketal with pivaloxymethyl iodide (POM-I) and deprotection [47]. In-depth studies evidenced that **45** had great stability in Dulbecco’s modified eagle medium complete (cDMEM) (Table 5) and efficient uptake inside cells wherein it was selectively hydrolyzed to the corresponding 3-*O*-POM-quercetin, with no trace of other metabolites (i.e., 7-*O*-POM-quercetin or quercetin) being detected.

Significant cytostatic activity of **45** was observed in MCF-7, HCT-116, and DU-145 cancer cell lines, as compared to quercetin which gave no inhibition of cell proliferation. Importantly, **45** displayed a cancer cell specific cytostatic effect, and no action was demonstrated on normal human diploid fibroblast (HS 27) cell line. Mechanistically, it has been proposed that the quercetin-POM conjugate **45** operates via a different pathway against quercetin, with cell cycle arrest taking place in the G0/G1 phase.

In a proof-of-concept study, Chong et al. demonstrated that 7-*O*-POM-quercetin (**46**, Figure 5) was able to reverse MDR in drug-resistant MES-SA/Dx5 cells derived from the drug-sensitive human uterine sarcoma (MES-SA) cell line (Table 6) [48,49].

Mechanistically, it was evidenced competition of **46** with verapamil binding to the P-glycoprotein (P-gp), which is a major MDR target. Moreover, **46** proved to be considerably more potent than quercetin and as active as verapamil in inhibiting the drug efflux mediated by P-gp.

Importantly, **46** evidenced accumulation inside MES-SA/Dx5 cells wherein it persisted along with its hydrolyzed product quercetin and quercetin metabolites (glucuronide and sulfate) for more than 48 h. As a result, the intracellular levels of **46** were adequately high to elicit the increased MDR-reversal effect as compared to quercetin.

Suitably protected quercetin derivatives were reacted with iodomethyl isopropyl carbonate (POC-I)/K_2_CO_3_ system in either DMF or DMF/acetone mixture and the compounds obtained were then deprotected to afford the quercetin conjugates **47**–**49** (Figure 5) bearing an isopropyloxycarbonylmethoxy (POC) group at 3-OH and/or 7-OH [50]. These species were deeply studied with regard to solubility, stability, permeability and intracellular metabolism. Compounds (**47**) and (**49**) were poorly soluble in phosphate-buffered saline (PBS) differently to **48** which proved to dissolve well in the same medium even at high concentrations. Anyhow, complete dissolution of all derivatives was observed in cDMEM (up to 100 μM concentration). With regard to stability, it has been demonstrated that quercetin-POC conjugates were almost as stable as the quercetin-POM derivatives [46,47]. All compounds featured high stability in PBS (*t*_1/2_ >96 h) (Table 7), while either decomposition or hydrolysis occurred in cell-free culture medium. Thus, the 3,7-bis-*O*-POC derivative **49** was hydrolyzed into 3-*O*-POC compound **48**, whereas the 7-functionalized analog **47** gave rise to decomposition and/or hydrolysis to the mother quercetin. Among the series, 3-*O*-POC **48** showed the best stability profile in term of resistance to both decomposition and hydrolysis.

Besides, membrane permeability assays assessed that the 7-conjugated derivative **47** behaved as quercetin, while 3-*O*-POC-quercetin **48** was the less permeable. In any case, the permeability of **48** is worthwhile noting as the corresponding 3-*O*-POM conjugate was totally impermeable [46]. Remarkably, no data could be obtained for **49** due to its low solubility in PBS at the concentration (25 μM) used for the membrane permeability test.

Cell-line-dependent hydrolytic and metabolic profiles were observed for quercetin derivatives **47**–**49**. On the one hand, they were smoothly converted to quercetin and its metabolites in MCF-7 cell line, with quercetin glucuronide being predominantly formed in all cases, according to literature data [51]. It should be highlighted that these results were completely different from those observed for the quercetin-POM analogs, which have been shown to be less prone to both intracellular hydrolysis and metabolism [46,47]. In addition, 3-*O*-POC-quercetin **48** was easily hydrolyzed and metabolized contrary to 3-*O*-POM-quercetin and 3,7-bis-*O*-POM-quercetin **45** [46,47].

On the other hand, **47**–**49** underwent slow hydrolysis and low metabolism in HCT-116 cells. In particular, 7-*O*-POC-quercetin (**47**) hydrolyzed to quercetin but neither of its metabolites was detected, while 3-*O*-POC-quercetin (**48**) proved to be very stable (up to 12 h) giving no trace of quercetin. This metabolic profile was also typical of 3,7-bis-*O*-POC-quercetin (**49**), but its hydrolysis hastened (*t*_1/2_ ≅ 3 h) in relation to cell-free medium (*t*_1/2_ = 24 h, Table 7). In this case, 3-*O*-POC-quercetin (**48**) was formed as the exclusive hydrolysis product.

Cytotoxic activities of **47**–**49** were strictly related to their stability properties. As a matter of fact, low antiproliferative effects against MCF-7 cells were observed for the POC-protected quercetins likely due to enhanced passive transport, intracellular hydrolysis, and metabolism. More precisely, compounds **47**–**49** were as active as quercetin. On the contrary, **47** and **49** displayed higher cytotoxicity than quercetin in HCT-116 cells, with **49** being more effective than **47**. Given the slow hydrolysis and metabolism of **47** and **49** in HCT-116 cells, both these compounds and their hydrolyzed products were present at concentrations high enough to enhance cytotoxicity. Importantly, **48** was not cytotoxic at all.

In addition to alkyl halides, 2,3-dichloro-1,4-naphtoquinone has been employed to alkylate quercetin using *N*,*N*-diisopropylethylamine as the base giving chloronaphtoquinone quercetin (**50**, CHNQ), which is depicted in Figure 5. This compound featured a 3-fold higher cytotoxicity than quercetin in colorectal (HCT-116 and HT-29) cancer cells (Table 8), and strong apoptosis-inducing effects, too, have been observed [52].

Furthermore, likely due to the presence of the 1,4-napthoquinone framework, treatment of cells with **50** resulted in a potent generation of oxidative stress leading to reactive oxygen species (ROS)-induced autophagy in vitro. In particular, the authors highlighted that complete autophagy occurred in HCT-116 cells, while incomplete autophagy took place in the HT-29 ones. Herein, CHNQ promoted LC3 lipidation, with the formation of acidic vacuoles being not observed.

It should be pointed out that conversion of quercetin into an isomeric chloronaphtoquinone derivative has been previously reported by Danihelová et al. [53]. This structural modification led to lower the antioxidant properties of quercetin, but enhanced the cancer cell inhibitory activities. However, the chloronaphtoquinone derivative also featured cytolytic effects towards non-cancer murine fibroblast (NIH-3T3) at a concentration of 100 μM, but the total degeneration of cancer cells (HeLa) took place at lower concentrations (50 μM).

#### 2.1.2. *O*-Alkylation and C-4 Carbonyl Group Modification

The *O*-alkylation of quercetin has been conveniently coupled with the bioisosteric conversion of the C-4 carbonyl group into the corresponding thiocarbonyl or selenocarbonyl moieties. So, synthetic routes involving suitable methylation of quercetin followed by either oxygen/sulfur or oxygen/selenium exchange and deprotection have been carried out to provide the sulfur- and seleno-compounds **51**–**54**, which are shown in Figure 6.

Martins et al. reported the synthesis of analogs **51**–**53** via chalcogenation of the quercetin-derived 3,3′,4′,7-*O*-tetramethyl compound (**19**, Figure 4) [54]. All the compounds have been tested on nine human cancer cell lines, namely melanoma cells (A375), colorectal adenocarcinoma cells (HCT-15), pancreatic adenocarcinoma cells (BxPC3), MCF-7 cells and the multidrug-resistant variant MCF-7/ADR, cervical adenocarcinoma cells (A431) and the corresponding cisplatin-resistant one (A431/Pt), cisplatin-sensitive and cisplatin-resistant ovarian adenocarcinoma cells (2008 and C13*). For the purpose of comparison, the same cancer cell lines were used in parallel experiments with quercetin, **19**, and cisplatin.

Thus, the selenium compound **51** showed a 9-fold and 3-fold higher cytotoxicity than quercetin and cisplatin, respectively, while **19** proved to be 3-fold less cytotoxic than quercetin (Table 9).

This result suggested that the selenocarbonyl moiety rather than the flavonoid core was responsible for the observed biological effects. On the contrary, unreproducible data were observed for the sulfur-containing derivatives **52** and **53** as a result of their low stability in solution. Furthermore, protected selenoquercetin **51** was able to overcome cisplatin-resistance due to comparable cytotoxic action against both the cisplatin-sensitive and cisplatin resistant cell lines. Remarkably, preliminary studies aimed at understanding the mechanism of cytotoxicity in MCF-7 cells evidenced that **51** hampered thioredoxin reductase (TrxR) activity, whilst it lacks efficacy to affect the glutathione peroxidase (GPx)/glutathione reductase (GR) redox system.

Exhaustive *O*-methylation of quercetin to the 3,3′,4′,5,7-*O*-pentamethyl derivative (**20**, Figure 4) followed by thionation gave access to compound **54**, which was eventually deprotected yielding **53** [55,56]. It has been shown that both compounds possess in vitro antiangiogenic and antiproliferative properties [55]. As a matter of fact, they were able to inhibit the migration of human umbilical endothelial vascular cells (HUVECs) promoted by the vascular endothelial growth factor (VEGF). However, the antiangiogenic activity of thiocarbonyl compounds **53** and **54** was inferior to that of both quercetin and **20**, with **54** being more active than **53**. Besides, **53** and **54** had antiproliferative activity towards MCF-7 cancer cell line and the doxorubicin-resistant variant MCF-7/DX, but much higher concentrations (approximately a 10 to 100-fold increase) were required compared to those determining the antiangiogenic action. In particular, the observed antiproliferative effects were in the order quercetin > **53** > **20** > **54**. Overall, derivative **20** was found to be the top of the line as it optimally balanced the high antiangiogenic activity with minimal toxicity. Further antiproliferative studies using breast cancer cell line MDA-MB-231 evidenced that the 4-thio compounds, and in particular **53**, had greater effects than **20** and the parent quercetin [56]. However, **53** did not reach the IC_50_ at 10 μM. Moreover, **20**, **53**, and **54** proved to be less active than quercetin towards both MCF-7 and MCF-7/DX cell lines, with the lowest activity being observed in the latter. This result was supposed to depend on the fact that these compounds are likely to act as substrates of the P-gp efflux pump, which is known to be over expressed in the MCF-7/DX cell line. It is worthwhile noting that in all cases no stability problems of **53** were pointed out, in contrast with others [54].

#### 2.1.3. Replacement of Catecholic Hydroxyl Groups

Along their studies aimed at separating the biological activities of quercetin from its antioxidant features, Cho et al. undertook the structural modification of quercetin by replacing the catechol hydroxyl groups with bioisosteric fluorine atoms [57]. Accordingly, a fragmentation-acylation-ring closure strategy was applied to convert quercetin into the corresponding 3′,4′-difluoro derivative **55** (Figure 7).

As anticipated, **55** proved to be stable against oxidative decomposition. Most likely, the non-radical-producing fluorine moieties prevent the production of *o*-quinone and *p*-quinone methide species that are typically formed upon oxidation of quercetin [58]. Nevertheless, **55** was as active as quercetin in the MCF-7 cell line, and a modest effect in hepatoma (Huh-7) cell line was also observed. Besides, it has been evidenced that the profile of early apoptotis of **55** resembles that of quercetin, albeit the latter induces cells in late apoptotic-necrotic stage more effectively.

#### 2.1.4. *O*-Acylation

Introduction of ester and urethane moieties into the quercetin scaffold produced compounds with improved cytotoxic action. This is likely due to a better bioavailability as a possible result of lipophilization [53,59,60]. During the period 2012–2016, synthesis and anticancer activities of *O*-acylated quercetin derivatives **56**–**63** (Figure 8) were reported.

Danihelová et al. prepared fifteen quercetin-derived compounds through condensation or selective protection reactions followed by acylation with acyl chlorides [53]. Among the series, pentaacetyl quercetin (**56**), di(tetraacetylquinoyl)quercetin (**57**), and tri(diacetylcaffeoyl)quercetin (**58**) exhibited the highest cytotoxicity towards HeLa cells and the non-cancerous cell line NIH-3T3. Notably, all these compounds were more effective than quercetin regardless of cell type.

Conversion of quercetin into the corresponding diphenylmethylketal followed by esterification with aspirin at the 3- and 7-OH gave access to quercetin aspirinates **59**–**61** showing higher cytotoxic effects against liver (HepG2) and promyelocytic leukemia (HL-60) cancer cells than quercetin [61].

Results of the cytotoxic activities of compounds **56**–**61** are reported in Table 10.

It has been previously demonstrated that quercetin amino-acid conjugates possess properties (i.e., water solubility, hydrolytic stability, cell permeability) superior to those of quercetin [62]. Based on this result, Kim et al. prepared six quercetin derivatives appended with alanine or glutamic acid residues at 3-*O* and/or 7-*O* sites [63]. Key steps in the synthetic strategy concerned the selective protection/deprotection of quercetin hydroxyl groups and the coupling of the intermediates obtained with suitably protected alanine and glutamic acid compounds.

The quercetin-amino acid analogs were tested for their cytotoxicity and ability to modulate MDR using the MES-SA cell line and the corresponding drug-resistant MES-SA/Dx5, that is known to overexpress P-gp [49]. At the concentration levels applied for MDR modulation (5 μM), the quercetin-amino acid conjugates showed no cytotoxic action in MES-SA cell line, likewise quercetin. Importantly, addition of the latter or the quercetin-amino acid derivatives did not affect the cytotoxic properties of a given anticancer agent, similarly to verapamil which has been used as the positive control. With regard to MES-SA/Dx5 cell line, MDR-reversal activity of the quercetin-amino acid compounds was strictly dependent on either the nature or position of the amino acid moieties. The 7-*O*-functionalized compounds in the series displayed the most potent effects, and the best results were evidenced for the quercetin-7-*O*-glutamic acid conjugate **62**. As depicted in Table 11, this compound proved to be 30.5-fold more active (fold-reversal, FR= 58.6) than quercetin (FR = 1.9) in reversing MDR towards doxorubicin, and was potent as much as the doxorubicin-resistance reversing drug verapamil (FR = 68.0). Additionally, **62** showed 13.8–14.8-times enhanced MDR-reversal effects against other anticancer drugs, including actinomycin D, vinblastine, and paclitaxel.

It is worthy of note that the MDR modulatory activity of **62** was not dictated by the stereochemistry of the amino acid promoiety. As a matter of fact, the synthetically prepared enantiomer of **62** was a potent MDR modulator (FR = 52.2), though less than **62** itself. Moreover, flow cytometric analysis and P-gp ATPase test evidenced that **62** inhibited the drug efflux by P-gp, and stimulated ATPase activity of P-gp by interaction with its drug-binding site, respectively.

Evaluation of the physico-chemical properties of compound **62** highlighted that the presence of the glutamic acid residue accounted for enhanced solubility, stability, and cellular uptake of quercetin. Indeed, **62** was markedly soluble in aqueous medium up to 400 μM, while quercetin solubility harshly dropped at concentration >100 μM. In comparison with quercetin, compound **62** possessed high stability both in PBS (*t*_1/2_ > 72 h) and Roswell Park Memorial Institut (RPMI)-1640 complete culture medium (cRPMI), with decomposition occurring only after 9.3 h (Table 12).

Importantly, the intracellular level of **62** remained adequately high for a prolonged period of time (6–24 h) due to slow metabolism to quercetin and quercetin metabolites (i.e., quercetin glucuronide and quercetin sulfate) that co-existed with **62**. This result may profile the use of **62** as a safe MDR modulator given the riskless nature of quercetin. Quercetin was also reacted with *n*-butyl-isocyanate to obtain the 3,3′,4′,7-*O*-tetraacylated derivative **63**. This compound proved to inhibit the proliferation of MCF-7 cells in vitro [64], with the IC_50_ value obtained being 36 μM compared to 128 μM for quercetin.

### 2.2. Functionalization of A- and B-Rings

The quercetin framework has been suitably modified by the insertion of sulfonate, prenyl, aminomethyl, and phenylethenyl appendages into A- and B-rings to provide derivatives **64**–**71** which are listed in Figure 9.

Zhang et al. reported the sulfonation of quercetin with concentrated sulfuric acid (H_2_SO_4_, 98%) to provide the water-soluble quercetin-5′,8-disulfonate sodium (**64**) [65]. This compound had higher antiproliferative/cytotoxic activity than quercetin against human colon (LoVo) and breast (MCF-7) cancer cells (Table 13). In particular, it has been found that **64** was less sensitive to LoVo cells as compared to the MCF-7 ones. As regards the cancer cell growth inhibition, it has been demonstrated that **64** acted like quercetin in both cancer cell types. Indeed, it decreased the cell cycle progression at G0/G1 phase and induced growth arrest at S-phase. In addition, **64** proved to be a more powerful pro-apoptotic agent than quercetin, with cell apoptosis taking place via a ROS-dependent pathway.

Recently, it has been shown that **64** could be a possible chemopreventive and chemotherapeutic agent for liver diseases, due to its potent hepatoprotective activity [66].

Along their studies towards the synthesis of poinsettifolin A (**65**), a geranylated flavonol isolated from *Dorstenia poinsettifolia* var. *angusta* Engl. (Moraceae) [68], Escobar et al. condensed quercetin with citral under microwave irradiation to obtain the benzopyran intermediate **66** which could be possibly converted into the natural target by C-6 prenylation [69]. Unfortunately, this operation failed to provide *C*- and/or *O*-prenylated derivatives and gave degradation products of **66** depending on the reaction conditions. As a consequence, an alternative route to **65** was developed via prenylation of appropriately protected quercetin followed by condensation with citral at C-8.

Biological evaluation studies on both **65** and **66** evidenced that the latter had potent cytotoxic action against lung cancer (A549, IC_50_ = 0.022 μM) and leukaemia (Jurkat, IC_50_ = 0.005 μM) cells, with the adherent cancer epithelial A549 cells being less sensitive to **66** in comparison to Jurkat cells, which grow in suspension. On the contrary, **65** was totally inactive in the same cell lines. Aminomethyl residues were introduced on the C-6 site of quercetin by reaction with amines and formaldehyde providing the benzopyran derivatives **67**–**69**, which were biologically essayed for in vitro cytotoxicity or RAC-alpha serine/threonine-protein kinase Akt1 inhibitory activity [70]. In detail, the cytotoxic action of **67**–**69** was tested against HL-60, OVCAR-8, PC-3, and HepG2 human cancer cell lines using the known Akt inhibitor HT-89 as a positive control [71]. These compounds displayed structure-dependent effects, with the *N*-methylbenzyl substituted analog **69** showing a better activity than the morpholine- and piperidine-containing ones (Table 13). Furthermore, favored Akt1 inhibitory action was observed for compounds **67** and **69**. Basing on these data, molecular dynamics simulation and molecular docking studies were carried out to have insights into the binding mode of the model substrate **69** to Akt1. The results obtained revealed that the Akt1 binding pocket enclosed **69**, with π–π stacking interactions involving the benzyl residue, while the catechol and 5-OH groups participated in hydrogen bonding. Even so, a definitive opinion on the activity of compounds **65**–**69** cannot be given as the authors did not furnish any comparison with quercetin.

Acid-promoted reaction of quercetin with phenylacetaldehyde yielded the isomeric derivatives **70** and **71** which featured moderate cytotoxic activity on the HepG2, SMMC-7721 and QGY-7703 liver cancer cell lines (Table 13) [67].

### 2.3. Metal Coordination

Application of metal-based drugs for therapeutic purposes has attracted much attention since the introduction of cisplatin into clinical practice for anticancer therapy [72]. However, tolerance issues and resistance of tumors to cisplatin hardly hamper its clinical success [73]. So, intensive studies have been made to obtain new platinum-based alternatives as well as organometallic complexes containing a metal ion other than platinum [74], showing that the pharmacological effects depend on the metal ion, the organic scaffold, and DNA binding site [75]. In particular, the binding of transition metal complexes with DNA has drawn particular consideration [76], as they could find possible use in cancer therapy [77].

**Figure 10:**
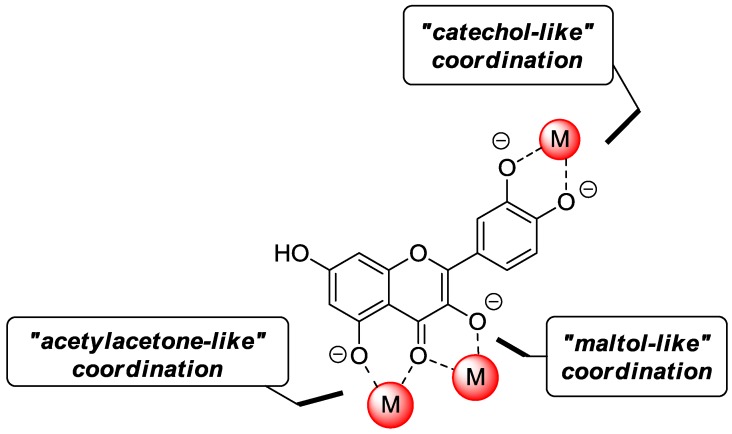
Possible coordination modes of quercetin with metal ions (M).

Due to the presence of hydroxyl and keto groups, quercetin is a very effective metal chelator. As depicted in Figure 10, three coordination modes are possible using 3-hydroxy-4-keto (“maltol-like” coordination), 5-hydroxy-4-keto (“acetylacetone-like” coordination) and cathecol (“catechol-like” coordination) functionalities.

In the recent past, quercetin-metal complexes have been the subject of intensive research proving that chelation may produce biological activities depending on the coordinating metal*.* Importantly, quercetin-metal complexes show better pharmacokinetic properties in vitro due to a well-defined geometric spatial orientation in the active site resulting from incorporation of the metal ion [78]. 

**Figure 11:**
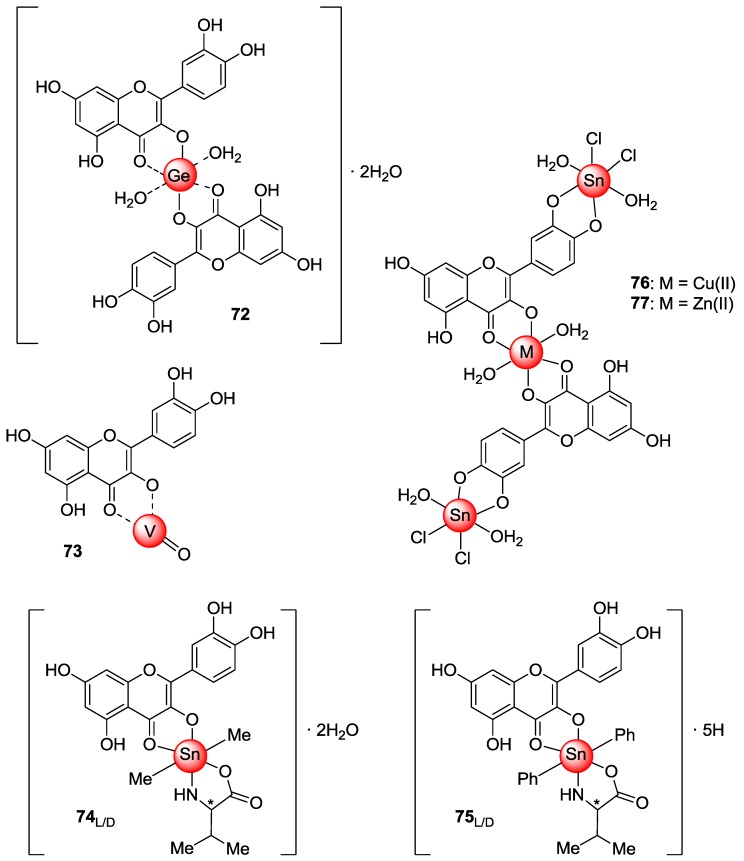
Structures of quercetin-metal complexes **72**–**77**.

Over the last five years, monometallic complexes of quercetin with Ge(IV)*,* V(IV)O, and Sn(IV), as well as heterobimetallic quercetin-Cu(II)/Zn(II)-Sn_2_(IV) derivatives (Figure 11) have been prepared and tested for their activity on diverse human cancer cell lines.

The quercetin-Ge(IV) complex **72**, obtained by reacting quercetin with germanium dioxide in basic medium [79], proved to possess significant in vitro cell growth inhibitory activity in human lung (SPC-A-1), human esophageal (EC9706), HeLa, and PC-3 cancer cell lines (Table 14), but these data lack of a comparative relation to quercetin. Compound **72** showed apoptosis-inducing effects, too. Basing on previous results reported by Tan et al. [80], these activities were supposed to arise from the planarity of quercetin ligand which may assist the intercalation of the metal complex into DNA thereby inducing its oxidative damage.

Treatment of an ethanolic solution of quercetin with an aqueous solution of vanadyl dichloride at pH 4 afforded oxovanadium(IV) complex [VO(Quer)_2_EtOH]*_n_* [81], which is schematically represented as **73** in Figure 11. This species featured better cytotoxic activity than quercetin and the oxovanadium(IV) cation against four human breast cancer cells, namely MDA-MB231, MDA-MB468, T47D and SKBR3, with the best effects being observed in the MDA-MB231 cell line (Table 15).

Remarkably, normal breast epithelial cells were not affected by compound **73**. Deep studies revealed that cancer cell death occurred via a mechanism that is different from that of oxovanadium(IV) cation and free quercetin. Most likely, intercalation is responsible for the activation of a mitochondrial route going along with enhanced DNA damage. Indeed, it has been observed that incubation of cancer cells with **73** caused a scanty intensification of ROS production, caspase activation and histone phosphorylation in addition to a small decrease of membrane potential.

The synthesis of L- and D-valine-quercetin diorganotin(IV) complexes **74**_L,D_ and **75**_L,D_ has been achieved by triethylamine-mediated reaction of quercetin with either L- or D-valine in the presence of dimethyltin and diphenyltin dichloride, respectively [82]. The metal-complexes belonging to the L-series exhibited notable in vitro biological actions anyhow higher than those of the corresponding D-enantiomers. Besides the high binding affinity to DNA, **74**_L_ and **75**_L_ showed good cytotoxicity against HepG2, MCF-7, HeLa, and MIA-Pa-Ca-2 (pancreas) cancer cells (Table 16) as well as significant human Topoisomerase I inhibition.

In particular, the diphenyl substituted derivative **75**_L_ possessed a better activity profile as compared to the dimethyl substituted analog **74**_L_. Unfortunately, the bioactivity data of **74**_L_ and **75**_L_ were compared to those of adriamycin, while a correlation with quercetin was totally missing. It is worthwhile noting that the results observed are in line with those reported for other organotin(IV) complexes [83,84,85]. It has been proposed that cell growth arrest and resulting apoptotic cell death may arise from inhibition of topoisomerase I-DNA or ‘cleavage complex’ formation as distinct from classical anticancer drugs (e.g., camptothecin) targeting topoisomerase I*.* Moreover, several factors have been accounted for the high cytotoxicity of the complexes. These include the presence of both the electron rich phenyl moiety and the organotin apoptotic template, with the vacant coordination sites on Sn(IV) center being available to form relatively stable ligand-Sn bonds (e.g., Sn–N, Sn–O and Sn–C) that cause slow hydrolytic decomposition [86]*.*

The heterobimetallic quercetin-Cu(II)/Sn_2_(IV) complex **76** and the corresponding Zn(II)/Sn_2_(IV) analog **77** were respectively synthesized by treatment of tin tetrachloride with monometallic quercetin-Cu(II) and quercetin-Zn(II) complexes, in turn obtained upon reaction of quercetin with either copper or zinc nitrate, each in order [87]. Compounds **76** and **77** have been identified as potential metal-based anticancer drugs due to in vitro DNA binding and cleavage properties as well as topoisomerase I inhibitory activity.

In-depth studies revealed that both complexes featured a dual mode of binding to DNA. That is to say, the Sn(IV) ions coordinate the oxygen atoms of the phosphate backbone via electrostatic interactions, while Cu(II)/Zn(II) ions exhibit coordinate covalent binding to N-3/N-7 positions of the nucleobases. In addition, hydrogen bonding interactions occur between the functional groups of DNA nucleobases and quercetin hydroxyl moieties thereby providing possible site-specific molecular recognition in cells.

Moreover, **76** and **77** were able to promote both single- and double-stranded DNA cleavage. In particular, it has been shown that complex **76** acted through a ROS-induced oxidative pathway, whereas **77** featured a hydrolytic cleavage route.

Importantly, **76** proved to be a catalytic inhibitor of topoisomerase I, with different modes of action being proposed with regard to this activity. These include (i) prevention of the enzyme to DNA binding, (ii) inactivation of the enzyme, and (iii) stabilization of the catalytic enzyme-DNA intermediate.

Speaking of cytotoxicity, both **76** and **77** proved to be active against the human carcinoma cells PC-3, HL-60, HCT-15, HeLa, Hop62, U373MG (central nervous system), and A2780 (ovarian). In particular, **77** revealed a smaller activity by comparison with **76**, which showed a similar degree of potency as the antitumor drug adriamycin (Table 17). In any case, a comparative relation of the activity of **76** and **77** to that of quercetin has not been given.

Interestingly, Dell’Anna et al. could obtain a quercetin-containing triphenylphosphane Pt(II) complex, but difficulties in getting it pure during its synthesis discouraged biological studies [88].

## 3. Structure-Activity Relationship

Comparison of the activities of quercetin and its modified forms led to identify structural-activity (SAR) relationships that mainly depend on the positions and nature of the substituents. In the series of the *O*-alkylated analogs either length or steric hindrance of the alkyl chain incorporated into the oxygen atoms influenced cancer cell growth inhibition. Activity was retained in the presence of a methoxy group at 4′- and/or 7-positions, a 3′,4′-bis-OMe functionalization improved the anticancer effects, and insertion of a third methoxy moiety at C-7 produced more potent actions [40]. Conversely, methylation at 3,3′,4′,7 hydroxyl groups caused a dramatic reduction of activity [54]. Substitution with either a propyloxy group at C-3 or an ethoxy one at C-4′ did not change inhibitory activities, whereas potency raised in the presence of two *n*-butyloxy residues at 3,7 or 4′,7 sites. This behaviour marked out the 3,7-dimethoxylated compound, too [41,42]. Functionalization of C-3, C-4′ and C-7 phenolic hydroxyl groups with linear long (propyl, butyl, pentyl, hexyl) or hindered (isopropyl, isopentyl) alkyl groups aroused a great drop of activity, which was slightly increased for both the 3,4′,7-triethoxy and 3,4′,7-trimethoxy substituted derivatives. By contrast, 3,3′,4′-*O*-triethylquercetin was as active as quercetin [42]. Eventually, linking butyl and geranyl chains at 7-OH produced much stronger antiproliferative effects [44,45].

Conjugation with the hydrolysable POC group resulted in tumor cell-specific cytotoxic activity, as in the case of 7-*O*-POC and 3,7-bis-*O*-POC functionalized analogs. These compounds displayed higher cytotoxicity than quercetin in HCT-116 cells, and the 3,7-bis-*O*-POC form was more active than the 7-substituted one [50].

The co-occurrence of a C-4 selenocarbonyl residue and methoxy substituents at 3,3′,4′,7 positions led to a dramatic increase of cytotoxicity [54]. Also, the unprotected thionated analog proved to possess greater antiproliferative effects than the mother flavone [56]. These results indicated that the chalcogenated carbonyl function rather than the flavonoid framework seemed to determine biological activity. On the contrary, replacement of catecholic hydroxyl groups with bioisosteric fluorine atoms did not produce remarkable results [57]. As far as *O*-acylated derivatives are concerned, generally higher anticancer activities than quercetin were observed regardless of the position and the nature of acyl groups [53,61,63,64].

Finally, functionalization of A- and B-rings and complexation with metals gave rise to a number of new analogs with marked cytotoxic action. Unfortunately, a detailed SAR analysis is not possible as comparison with quercetin has not been reported in all but two cases showing that double sulfonation at 5’ and 8 positions as well as complexation with oxovanadium(IV) cation were suitable strategies to improve quercetin anticancer properties [65,81].

## 4. Conclusions

Quercetin is a valuable biologically active compound with well-ascertained anticancer activity, however its unfavorable physico-chemical/pharmacokinetic profile as well as potential in vivo toxicity impede clinical applications to a large extent. Scientists have selected to tackle this problem by appropriately modifying the quercetin scaffold so as to obtain structural analogs with possibly better bioactivity, solubility, bioavailability, metabolic stability, and reduced toxicity. Over the five years from 2012 to 2016, a number of synthetic quercetin derivatives have been prepared through chemical manipulation of the phenolic hydroxyl groups and/or C-4 carbonyl moiety, functionalization of A- and B-rings, and complexation with metals. It does seem worthy of attention that the compounds obtained have been mainly studied for their in vitro effects on a range of cancer-derived cell lines, but there were just four papers reporting findings on physico-chemical and pharmacokinetic properties [45,47,50,63]. Though scanty, these data suggest that the conjugation of quercetin with a proper promoiety is pivotal in obtaining analogs with enhanced solubility and stability. As summarized in Table 18, marked aqueous solubility (up to 400 μM) was observed for the quercetin amino-acid conjugate **62**, while excellent stability profiles labeled 3,7-bis-*O*-POM-quercetin (**45**) and the 3-*O*-POC derivative **48**, with the former suffering hydrolysis to the corresponding 3-*O*-POM analog after 100 h. Similarly to **45**, 3,7-bis-*O*-POC-quercetin (**49**) proved to be more stable than quercetin, however **49** was more prone to hydrolysis than **45**.

As far as the anticancer effects of modified quercetins are concerned, the reported activities were mainly higher than quercetin. However, some confusing data have been also found as was the case with HTS-based studies [40,41]. As discussed by Baell and Holloway [90], caution should be taken when interpreting HTS-derived bioactivity results, which could furnish false positives among the hit compounds for various causes [91]. Certainly, this perspective seems to be appropriate to look into the whole data collected in this review. In our idea, the publication policies that are recommended by the authors for screening-based manuscripts are often enough not taken into consideration [90], so the final interpretation of either SAR or biological results may be misleading.

Most likely, the catechol group of quercetin should negatively influence choice scaffolding. Nevertheless, taking into consideration the wide number of modifications under different conditions reported in this review, it might be possible to remove some doubts about the efficacy of quercetin as starting material.

**Figure 12:**
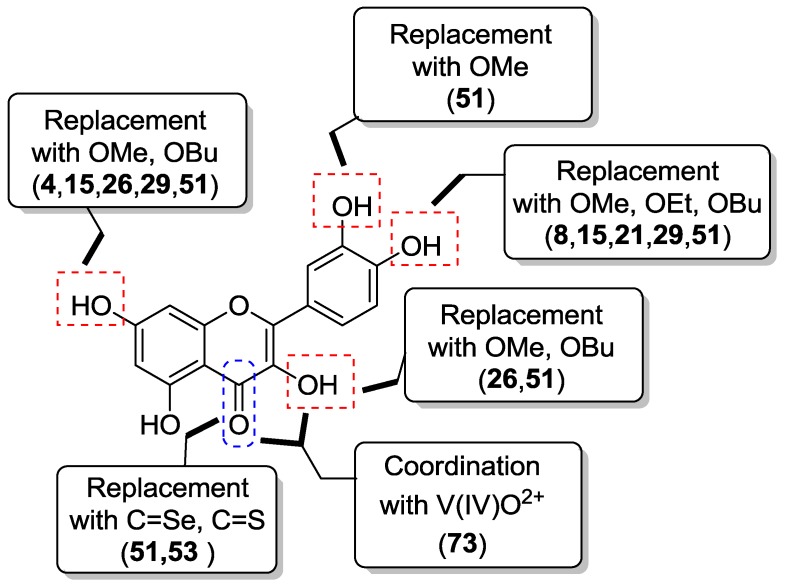
Structure modifications of quercetin leading to the most promising anticancer analogs.

Despite everything, we consider worthwhile to draw attention to the most promising analogs we described (Table 19). These include the compounds **1**, **4**, **8**, **15**, **21**, **26**, **29**, **51**, and **73** showing IC_50_ values inferior to 4–5 μM, which could be considered to determine in vitro cytotoxicity according to the criteria of the United States National Cancer Institute [92]. The structure modifications of quercetin leading to the most promising analogs are graphically depicted in Figure 12.

Drawing to a close, we hope that the data reported in this review will inspire further investigations into the anticancer activity of quercetin and its derivatives to identify optimal therapeutic candidates for effective cancer treatment.

## Figures and Tables

**Table 1:** Breast cancer resistance protein (BCRP) inhibition by compounds **19** and **20** compared to quercetin ^1^.

	1	19	20
Hoechst 33342	nd ^2^	0.540 ± 0.079	0.822 ± 0.169
Pheophorbide A	nd ^2^	0.570 ± 0.093	1.880 ± 0.240

^1^ IC_50_ ± standard deviation (SD) values as μM. ^2^ No inhibitory effect was observed up to 10 μM.

**Table 2:** Cytotoxicity data of the most representative *O*-alkylated quercetins compared to quercetin by high-throughput screening (HTS) method ^1^.

Cell Line	Compound						
	1	4	8	15	21	26	29
A549	6.20 ± 0.51	3.08 ± 0.10	2.63 ± 0.19	3.07 ± 0.02	1.24	1.13	2.06
H157	6.00 ± 0.47	3.31 ± 0.01	3.04 ± 0.02	3.45 ± 0.02	0.67	5.92	0.39
H460	9.62 ± 0.89	3.32 ± 0.02	4.45 ± 0.02	2.75 ± 0.01	1.03	3.05	3.16
1944	10.18 ± 1.11	4.25 ± 0.02	3.86 ± 0.02	2.86 ± 0.01	1.78	1.17	0.56
H266	16.87 ± 1.12	13.87 ± 1.16	7.57 ± 0.05	>50	2.85	8.92	15.92
Hop62	7.52 ± 0.58	6.87 ± 0.04	9.93 ± 0.78	12.11 ± 1.11	4.45	4.68	4.43
1299	13.60 ± 1.24	10.25 ± 0.88	21.97 ± 1.78	34.82 ± 3.21	1.77	5.05	4.54
292G	>50	27.95 ± 2.17	12.54 ± 1.10	40.72 ± 3.79	4.11	8.75	17.73
Calu1	23.58 ± 1.82	23.43 ± 1.76	24.95 ± 1.89	>50	1.50	5.46	6.21
1792	3.85 ± 0.45	14.73 ± 1.08	4.06 ± 0.03	3.36 ± 0.02	1.00	6.03	3.09
M4E	21.71 ± 1.87	10.82 ± 0.98	19.91 ± 1.95	30.69 ± 2.98	6.18	11.18	6.29
M14	12.77 ± 1.08	13.32 ± 1.76	14.85 ± 1.26	4.29 ± 0.02	0.94	3.47	9.35
LOX-IMVI	4.65 ± 0.28	>50	>50	>50	1.14	2.55	2.36
SKBR	16.71 ± 1.21	6.25 ± 0.03	7.97 ± 1.01	8.30 ± 0.54	3.19	4.58	4.39
Hela	3.56 ± 0.28	4.26 ± 0.02	1.81 ± 0.01	1.49 ± 0.01	1.20	6.09	0.87

^1^ IC_50_ ±SD or IC_50_ values as μM.

**Table 3:** Antiproliferative activities of derivatives **11** and **24**–**26** compared to quercetin in human prostate cancer cells ^1,2^.

Cell Line	Compound				
	1	11	24	25	26
PC-3	>100	22.27 ± 6.83	13.74 ± 1.62	17.68 ± 0.96	11.95 ± 1.12
DU145	>100	46.82 ± 3.69	12.59 ± 0.96	19.25 ± 2.21	18.63 ± 6.86
LNCaP	45.46 ± 1.31	13.23 ± 4.75	4.20 ± 0.96	6.42 ± 2.72	6.46 ± 1.10

^1^ IC_50_ ±SD values as μM. ^2^ After 72 h incubation.

**Table 4:** Cytotoxicity data of compounds **43** and **44** compared to quercetin ^1,2^.

Cell Line	Compound		
	1	43	44
MCF-7	343	43.5 ± 2.1 ^3^	22.6 ± 2.2 ^3^
		38.6 ^4^	20.2 ^4^
CaCo-2	340	66.8	43.7
NCI-H446	68.9		27.6
A549	77.2		29.5
MGC-803	80.6		25.4
SGC-7901	75.7		18.5

^1^ IC_50_ ±SD or IC_50_ values as μM. ^2^ After 48 h incubation. ^3^ Data taken from [44]. ^4^ Data taken from [45].

**Table 5:** Stability of compound **45** compared to quercetin in diverse media ^1^.

Medium	Compound	
	1	45
PBS	10 h	>24 h
cDMEM	<0.5 h	100 h ^2^

^1^ Half-time (*t*_1/2_) values. ^2^ Compound **45** is converted exclusively to 3-*O*-POM-quercetin. PBS: phosphate-buffered saline; cDMEM: Dulbecco’s modified eagle medium complete.

**Table 6:** Multi-drug resistance (MDR)-reversing activity of compound **46** compared to quercetin and verapamil ^1^.

Anticancer Drug	Modulator ^2^			
	None (IC_50_)	Verapamil (IC_50_/FR)	1 (IC_50_/FR)	46 (IC_50_/FR)
Doxorubicine	8.18 ± 0.01	0.12 ± 0.01/68.3	4.26 ± 0.32/1.9	0.34 ± 0.09/24.1
Actinomycin D	13.10 ± 0.34	0.23 ± 0.02/57.0	4.68 ± 1.00/2.8	0.41 ± 0.01/32.0
Vinblastine	12.25 ± 0.19	0.24 ± 0.01/51.0	4.90 ± 0.13/2.5	0.43 ± 0.06/28.5
Paclitaxel	10.53 ± 0.21	0.22 ± 0.04/47.9	4.66 ± 0.11/2.3	0.41 ± 0.04/25.7

^1^ IC_50_ ±SD values as μM (*p* < 0.01). ^2^ Used at 5 μM concentration. FR = fold-reversal (IC_50_ of anticancer drug alone/IC_50_ of anticancer drug combined with the modulator).

**Table 7:** Stability of quercetin-isopropyloxycarbonylmethoxy (POC) conjugates **47**–**49** compared to quercetin ^1^.

Medium	Compound			
	1	47	48	49
PBS	10 h	>96 h	>96 h	>96 h
cDMEM	<0.5 h	1 h	54 h	24 h ^2^

^1^ *t*_1/2_ values. ^2^ Compound **49** is hydrolyzed to **48**.

**Table 8:** Anticancer activities of compound **50** compared to quercetin ^1,2^.

Cell Line	Compound	
	1	50
HCT-116	117 ± 8.9	30.3 ± 1
HT-29	nc ^3^	23.21 ± 2.4

^1^ IC_50_ ±SD values as μM. ^2^ After 24 h incubation. ^3^ Proliferation did not change.

**Table 9:** Cytotoxicity of compound **51** compared to quercetin, **19** and cisplatin. ^1,2,3^.

Cell Line	Compound			
	1	51	19	Cisplatin
A375	25.13 ± 2.62	2.21 ± 1.07	69.72 ± 3.32	3.12 ± 1.13
HCT-15	16.35 ± 2.29	2.23 ± 1.01	68.11 ± 2.35	12.31 ± 1.26
BxPC3	24.12 ± 1.85	2.42 ± 1.19	>100	11.43 ± 1.29
MCF-7	20.90 ± 3.44	3.08 ± 1.98	>100	7.6 ± 2.49
MCF-7/ADR	22.15 ± 2.14 (1.1)	3.35 ± 1.58 (1.1)	>100	8.41 ± 1.22 (16)
A431	23.04 ± 1.07	3.11 ± 1.23	57.54 ± 2.28	1.62 ± 1.25
A431/Pt	34.37 ± 3.22 (1.5)	3.78 ± 1.52 (1.2)	82.25 ± 2.77 (1.4)	3.42 ± 1.08 (2.1)
2008	21.18 ± 1.84	2.09 ± 1.27	>100	2.17 ± 1.37
C13*	37.62 ± 3.82 (1.8)	2.29 ± 1.93 (1.1)	>100	22.26 ± 1.86 (10.3)

^1^ IC_50_ ±SD values as μM (*p* < 0.05). ^2^ After 72 h incubation. ^3^ Resistance factor (RF: IC_50_ resistant cell/IC_50_ parent cell line) values are reported in parentheses.

**Table 10:** Cytotoxic data of analogs **56**–**61** compared to quercetin ^1^.

Cell Line	Compound						
	1	56	57	58	59	60	61
HeLa	35.5 ± 1.1 ^2^	29.6 ± 1.9 ^2^	19.5 ± 0.8 ^2^	16.5 ± 1.5 ^2^			
NIH-3T3	20.9 ± 0.9 ^2^	15.5 ± 0.7 ^2^	16.1 ± 0.4 ^2^	10.6 ± 0.1 ^2^			
HL-60	>100				68.71 ^3^	69.29 ^3^	>100 ^3^
HepG2	>100				54.22 ^3^	38.49 ^3^	55.80 ^3^

^1^ IC_50_ ±SD or IC_50_ values as μM. ^2^ After 72 h incubation. ^3^ After 48 h incubation.

**Table 11:** MDR-reversing activity of compound **62** compared to quercetin and verapamil ^1^.

Anticancer Drug	Modulator ^2^			
	None	Verapamil	1	62
Doxorubicine	8.20	0.12	4.26	0.14
Actinomycin D	0.13	0.23	4.68	0.34
Vinblastine	0.11	0.24	4.90	0.33
Paclitaxel	0.10	0.22	4.66	0.32

^1^ IC_50_ values as μM. ^2^ Used at 5 μM concentration.

**Table 12:** Stability of quercetin-glutamic acid conjugate **62** compared to quercetin ^1^.

Medium	Compound	
	1	62
PBS	10.3 h	>72 h
cRPMI	0.4 h	9.3 h

^1^ *t*_1/2_ values. PBS: phosphate-buffered saline. cRPMI: Roswell Park Memorial Institut (RPMI)-1640 complete culture medium.

**Table 13:** Activity data for compounds **64** and **67**–**71** compared to quercetin ^1^.

Cell Line	Compound						
	1	64 ^2^	67 ^3,4^	68 ^3,4^	69 ^3,4^	70 ^5^	71 ^5^
LoVo	40.2 ^2^	28.0					
MCF-7	30.8 ^2^	19.9					
HL-60			16.65 ± 1.5 (11.45 ± 0.9)	14.22 ± 1.1 (11.45 ± 0.9)	7.56 ± 0.4 (11.45 ± 0.9)		
OVCAR-8			>50 (7.74 ± 0.5)	>50 (7.74 ± 0.5)	16.69 ± 2.0 (7.74 ± 0.5)		
PC-3			42.74 ± 3.8 (9.49 ± 0.8)	32.28 ± 2.2 (9.49 ± 0.8)	15.45 ± 1.8 (9.49 ± 0.8)		
HepG2	22.16 ± 0.63 ^5,6^		12.50 ± 0.9 (10.17 ± 0.6)	18.57 ± 1.1 (10.17 ± 0.6)	7.20 ± 0.5 (10.17 ± 0.6)	>40	25.76 ± 1.12
SMMC-7721	>40 ^5^					>40	>40
QGY-7703	18.90 ± 0.48 ^5^					13.66 ± 0.90	17.70 ± 0.49

^1^ IC_50_ ±SD or IC_50_ values as μM. ^2^ After 48 h incubation. ^3^ After 72 h incubation. ^4^ Data for HT-89 in parentheses. ^5^ After 24 h incubation. ^6^ Data taken from [67] for comparison with activities of **70** and **71**.

**Table 14:** Cancer cell 72 h inhibition rate by **72** ^1^.

Cell Line	Concentration	
	10 μM	100 μM
EC9706	8.67 ± 5.16	35.12 ± 3.80 ^2^
PC-3	7.28 ± 4.18	29.72 ± 2.84 ^3^
HeLa	3.86 ± 3.85	33.72 ± 6.07 ^2^
SPC-A-1	5.04 ± 0.76	47.24 ± 2.09 ^2^

^1^ Growth inhibition (GI) as percentage (%) values. ^2^ *p* < 0.001. ^3^ *p* < 0.01.

**Table 15:** Cytotoxicity data of **73** compared to quercetin and oxovanadium(IV) cation ^1,2^.

Cell Line	Compound		
	1	73	V(IV)O^2+^
MDA-MB231	49.6 ± 6.0	10.2 ± 8.0	49.0 ± 2.5
SKBR3	25.8 ± 4.5	22.8 ± 7.6	95.7 ± 4.8
MDA-MB468	23.8 ± 5.2	7.4 ± 5.4	19.4 ± 2.0
T47D	81.5 ± 4.8	4.8 ± 7.6	>100

^1^ IC_50_ ±SD values as μM. ^2^ After 48 h incubation.

**Table 16:** Cytotoxicity data of **74**_L_ and **75**_L_^1^.

Cell Line	Compound		
	ADR	74_L_	75_L_
HeLa	<0.0184	<0.0166	<0.0144
MCF-7	<0.0184	<0.0166	<0.0144
MIA-Pa-Ca-2	<0.0184	<0.0166	<0.0144
HepG2	<0.0184	0.0757	<0.0144

^1^ GI_50_ values as μM. ^2^ ADR: adriamycin.

**Table 17:** Cytotoxicity data of **76** and **77** ^1^.

Cell Line	Compound		
	ADR	76	77
U373MG	<17.2	<8.7	42.5
PC-3	<17.2	<8.7	40.0
Hop62	<17.2	<8.7	36.5
HL-60	<17.2	<8.7	31.9
HCT-15	<17.2	<8.7	42.8
A2780	56.0	>69.6	41.1
HeLa	<17.2	<8.7	7.7

^1^ GI_50_ values as μM. ^2^ Compounds with GI_50_ < 8.7 are considered active.

**Table 18:** Summarized data on solubility and stability of quercetin analogs.

Compound	Solubility (Solvent)	Stability (Solvent)
**1**	198 μM (water) [89]	10 h (PBS); <0.5 h (cDMEM) [47,50] 10.3 h (PBS); 0.4 h (cRPMI) [63]
**43/44**	180 μM (DMEM) [45]	
**45**		>24 h (PBS); 100 h (cDMEM) ^1^ [47]
**47**		>96 h (PBS); 1 h (cDMEM) [50]
**48**		>96 h (PBS); 54 h (cDMEM) [50]
**49**		>96 h (PBS); 24 h (cDMEM) ^2^ [50]
**62**	up to 400 μM (aqueous) [63]	>72 h (PBS); 9.3 h (cRPMI) [63]

^1^ *t*_1/2_ value for hydrolysis of **45** to 3-*O*-POM-quercetin. ^2^ Compound **49** is hydrolyzed to **48**.

**Table 19:** Anticancer activity of the most promising quercetin analogs compared to quercetin ^1^.

Cell Line	Compound								
	1	4	8	15	21	26	29	51 ^2^	73
A549	6.20 ± 0.51	3.08 ± 0.10	2.63 ± 0.19	3.07 ± 0.02	1.24	1.13	2.06		
H157	6.00 ± 0.47	3.31 ± 0.01	3.04 ± 0.02	3.45 ± 0.02	0.67		0.39		
H460	9.62 ± 0.89	3.32 ± 0.02	4.45 ± 0.02	2.75 ± 0.01	1.03	3.05	3.16		
1944	10.18 ± 1.11	4.25 ± 0.02	3.86 ± 0.02	2.86 ± 0.01	1.78	1.17	0.56		
H266	16.87 ± 1.12				2.85				
Hop62	7.52 ± 0.58				4.45	4.68	4.43		
1299	13.60 ± 1.24				1.77		4.54		
292G	>50				4.11				
Calu1	23.58 ± 1.82				1.50				
1792	3.85 ± 0.45		4.06 ± 0.03	3.36 ± 0.02	1.00		3.09		
M4E	21.71 ± 1.87								
M14	12.77 ± 1.08			4.29 ± 0.02	0.94	3.47			
LOX-IMVI	4.65 ± 0.28				1.14	2.55	2.36		
SKBR	16.71 ± 1.21				3.19	4.58	4.39		
Hela	3.56 ± 0.28	4.26 ± 0.02	1.81 ± 0.01	1.49 ± 0.01	1.20		0.87		
A375	25.13 ± 2.62							2.21 ± 1.07	
HCT-15								2.23 ± 1.01	
BxPC3								2.42 ± 1.19	
MCF-7								3.08 ± 1.98	
A431								3.11 ± 1.23	
2008								2.09 ± 1.27	
T47D									4.8 ± 7.6

^1^ IC_50_ ±SD or IC_50_ values as μM. ^2^ After 72 h incubation.
